# Spontaneous Perforation of the Stomach

**Published:** 1862-06

**Authors:** W. H. Butler

**Affiliations:** Assistant Surgeon Michigan Volunteers


ART. V.—*Spontaneous Perforation of the Stomach, by* W. H. BUTLER, M. D., *Assistant Surgeon Michigan Volunteers.*

August, 1859.—Made a post mortem examination of the body of Mrs. M. M——, Seneca street, who had been rather intemperate for some time, but generally healthy. Dr. Newell was called on the 20th, and found her vomiting, and with intense pain in the abdomen; he supposed it a case of cholera morbus, and prescribed accordingly. The next morning, in the absence of Dr. N., Dr. Gould saw her, when she was evidently sinking, and she died about 8 P. M.

Examination 14 hours after, 10 A. M., August 22d. Externally the body very yellow, and the face had a palid, emaciated look, (the worn out appearance caused by severe pain.) Ends of fingers quite black, rigor mortis well marked; abdomen much distended in the epigastric and umbilical regions; hypostatic congestion well marked; the gums had a blanched look; eyes glazed; no perceptible smell arising from the body. Internally, some old adhesions on left lung—none on right; considerable serum in thoracic cavity; on the right side it is yellow—on the left, mixed with blood; heart—generally normal—rather fatty, contains a little blood in the left ventricle—none in right side. Abdomen: evidences of peritoneal inflammation abundant, patches of yellow lymph, and large quantities of serum in the abdominal cavity. The great omentum thickened and quite fatty; the small intestines are considerably injected with blood; the liver was of unusual size, and of a lighter yellow color than ordinary—weight 8½ pounds. Found under right lobe large patches of lymph, also between the lobes. Gall bladder large, and distended, with fluid. Stomach externally healthy; the internal surface had a chronic injected appearance, and the mucous coat rather thinned. It contained about two oz. of thick, viscid fluid, of a dark green color; the duodenal portion was less inflamed than the other. Found on the superior portion of the pyloric end and near the pylorus *a circular perforation* of the size of a ten cent piece; the external edges rather dark, but on the inner side they were thickened; the thickening seems to extend for half an inch around it. It looks much on the inner side as if cut or punched out by a sharp instrument. Spleen of usual size; in the lower part found a somewhat unusual occurrence. This was a spherical mass, more condensed than the rest of the organ, and set like the yolk of a hard boiled egg; it could be turned out like that; this was firmer than the surrounding tissue, and of the size of a pigeon's egg, embedded in the substance of the organ. In the abdomen, found as we estimated, a pint of serum, and great quantities of lymph. Kidneys, bladder and uterus healthy.

In my testimony on the case, I said: "There can be no doubt that death was due to the perforation of the stomach, thus following inflammation of the bowels and the great deposits of lymph that were seen. This also explains the vomiting with which she suffered. I have no doubt the perforation was due to natural cause, by ulceration. There does not seem to be any just grounds of suspicion of death from poisoning, or other than that by the perforation. The patient, we have learnt, has for years been addicted to spirit drinking, and this no doubt produced the chronic enlargement of the liver, and the great deposit of fat noticed on the intestines." This latter appearance was very peculiar, masses of fat hung like stalactites or fingers from the whole length of the intestines; these varied from one-fourth of an inch to one inch in length, and of variable widths.

Budd, in his work on "Diseases of the Stomach," alludes to this affection as *simple chronic* or *perforating ulcer*. Page 91 he says: "The outer coats of the stomach are always destroyed in less extent than the mucous coat, so that, when perforation occurs, the aperture seen from without is much smaller than the corresponding ulcer of the mucous membrane." Although a matter of no special importance, it is to be remarked, that the specimen now before me, preserved in alcohol, shows no difference, both edges being quite sharp, the outer, if either, the most corroded away. The margin, for a line or two being thickened on both sides, showing it to have been of long standing. I presume the ulcer commenced internally, progressing from within outward; even admitting that the external opening may have been the largest, this alone not being conclusive of its commencement externally. I argue this from the majority beginning in that manner, and from the intemperate habits of the woman. The ulcer, as in the majority of cases, was situated superiorly, at the lesser curviture.

Dr. Finnell reports, *N. Y. Medical Times*, June 8, 1861, p. 376, a case of this, occurring in a woman, aged 30, remarking it was the sixth specimen presensed to the New York Pathological Society; all but one were females. In two the opening was upon the posterior surface.

Perforation of the stomach is caused by ulceration, by poison, and from digestion; when due to the latter cause, generally occurring at the greater curvature. This comes from the liquids most frequently collecting there, and the mucous membrane is thinner. Although digestion of the stomach occurs at times in those dying from disease, it is most frequently noticed in those dying suddenly from violence, in health, when the temperature is warm, and particularly so as the result of fracture of the brain. Of the sexes, perforation from disease occurs oftenest in females; the most frequent age from 30 to 50, though met with from 16 to 60.

Death results in three ways in this affection: from hæmorrhage—the ulcer having destroyed the coats of an artery—generally the gastric or splenic; from peritonitis, the result of the escape of the contents of the stomach into the sac of the peritoneum; and from exhaustion. In the former case there may be several hæmorrhages, even years apart, the last proving fatal often in a few moments; in the second, the patient usually dies in from eighteen to thirty hours; this is the most frequent cause.

Perforation of the stomach is of great interest in connection with medico-legal investigations. In the present case the husband of the woman was suspected of having poisoned her, which the post mortem happily removed.

The symptoms commonly attack the individual suddenly, apparently while enjoying perfect health, and this circumstance if supported by others reflecting on any individual that may be in near relation to the deceased, is often sufficient to attach suspicions of the gravest moment. The differences may be briefly summed up as follows: In poisoning, by irritants, the pain usually comes on gradually, slowly increasing; vomiting usually severe, with purging, thirst, burning in the throat, &c. In poisoning by arsenic, (the most common) death takes place in from eighteen to thirty-six hours.

In perforation from disease, the symptoms come on suddenly; the attack commences with a sudden and severe pain in the abdomen, generally soon after a meal. Vomiting, if it exist at all, is commonly slight, and chiefly confined to what is swallowed. There is no purging; the bowels are generally *constipated.*

